# Uremic Toxin Clearance and Cardiovascular Toxicities

**DOI:** 10.3390/toxins10060226

**Published:** 2018-06-02

**Authors:** Robert D. Mair, Tammy L. Sirich, Timothy W. Meyer

**Affiliations:** 1 The Departments of Medicine, VA Palo Alto Healthcare System, 111R, 3801 Miranda Ave., Palo Alto, CA 94304, USA; tsirich@stanford.edu (T.L.S.); twmeyer@stanford.edu (T.W.M.); 2 Division of Nephrology, Stanford University, 777 Welch Road, Suite DE, Palo Alto, CA 94304, USA

**Keywords:** Uremia, cardiovascular disease, dialysis

## Abstract

Uremic solutes contribute to cardiovascular disease in renal insufficiency. In this review we describe the clearance of selected uremic solutes, which have been associated with cardiovascular disease. These solutes—indoxyl sulfate (IS), p-cresol sulfate (PCS), phenylacetylglutamine (PAG), trimethylamine-n-oxide (TMAO), and kynurenine—exemplify different mechanisms of clearance. IS and PCS are protein-bound solutes efficiently cleared by the native kidney through tubular secretion. PAG and TMAO are not protein-bound but are also cleared by the native kidney through tubular secretion, while kynurenine is not normally cleared by the kidney. Increases in the plasma levels of the normally secreted solutes IS, PCS, TMAO, and PAG in chronic kidney disease (CKD) are attributable to a reduction in their renal clearances. Levels of each of these potential toxins are even higher in patients on dialysis than in those with advanced chronic kidney disease, which can be accounted for in part by a low ratio of dialytic to native kidney clearance. The rise in plasma kynurenine in CKD and dialysis patients, by contrast, remains to be explained. Our ability to detect lower levels of the potential uremic cardiovascular toxins with renal replacement therapy may be limited by the intermittency of treatment, by increases in solute production, and by the presence of non-renal clearance. Reduction in the levels of uremic cardiovascular toxins may in the future be achieved more effectively by inhibiting their production.

## 1. Introduction

Uremic solutes likely contribute to the development of cardiovascular disease in CKD and dialysis patients. As glomerular filtration rate (GFR) decreases, renal clearance of many solutes decreases, and their plasma levels rise. Dialysis removes small uremic solutes from the blood. Plasma levels of many solutes, however, are higher in dialysis patients than in patients with advanced renal insufficiency.

The large number of solutes that accumulate in renal insufficiency makes it challenging to determine the toxicity of specific solutes. No controlled trials have demonstrated that altering levels of specific solutes improves cardiovascular outcomes in humans. In the absence of such studies, evidence for the cardiovascular toxicity of uremic solutes is based on observational, animal, and in-vitro studies. In this review, we consider representative uremic toxins that are cleared by diverse mechanisms. The solutes we discuss were selected to illustrate the different clearance mechanisms that may lead to varying degrees of solute accumulation in renal failure and prompt the use of different strategies to reduce plasma solute levels.

## 2. Uremic Solutes with Evidence of Cardiovascular Toxicity

Indoxyl sulfate (IS) is a prototypic uremic solute formed by host modification of a colon microbial metabolite. Colon microbes metabolize the amino acid tryptophan to indole, which is then oxidized and conjugated with sulfate in the liver. Observational studies have identified variable associations between plasma indoxyl sulfate and cardiovascular disease in renal insufficiency [1,2,3,4,5,6,7,8]. The variability of these findings may result from the difficulty of quantifying free, unbound indoxyl sulfate levels, from differences in study populations, and from inconsistent adjustment for the levels of other uremic solutes. Of note, no association between indoxyl sulfate levels and cardiovascular outcomes was noted in patients in the Hemodialysis (HEMO) trial [3]. Although the evidence from observational studies is inconsistent, animal and in-vitro studies lend plausibility to the cardiovascular toxicity of indoxyl sulfate [9]. Mechanistic studies have shown that indoxyl sulfate increases clotting in vascular smooth muscle cell preparations through activation of the aryl-hydrocarbon receptor and expression of tissue factor [10,11]. A recent study found that increasing indoxyl sulfate in mice to levels found in dialysis patients promoted clotting in response to vascular injury [12]. Additional mechanisms by which indoxyl sulfate may alter endothelial function are described by Burtey in this issue of Toxins.

p-Cresol sulfate (PCS) is also formed by host modification of a product of microbial amino acid catabolism. Colon microbes metabolize the amino acids tyrosine and phenylalanine to p-cresol, which is then conjugated with sulfate in the liver. Observational studies again offer inconsistent evidence of cardiovascular toxicity. Some have identified an association between PCS and cardiovascular events in stage 3–5 CKD [4,13,14] and dialysis patients [7,15,16], while others report negative results [3,6]. In-vitro studies have shown that PCS can cause endothelial dysfunction [17,18], though there is less experimental data supporting the pro-thrombotic effect of PCS than of IS [9].

Phenylacetylglutamine (PAG) is one more example of a solute formed by host modification of a product of microbial amino acid catabolism. Colon microbes metabolize phenylalanine to phenylacetic acid, which is then conjugated with glutamine in the liver. In a recent study, PAG was associated with mortality and time to first cardiovascular event in patients with CKD stage 1–5 [14]. However, no association between PAG level and cardiovascular outcomes was noted in patients in the HEMO trial [3].

Trimethylamine N-oxide (TMAO) is also formed by host modification of a colon microbial metabolite. In this case, however, the microbial metabolite is produced not from an amino acid but from dietary quaternary amines including choline, betaine, and carnitine [19]. Microbes produce trimethylamine, which is oxidized to TMAO in the liver. The association of plasma TMAO levels with cardiovascular outcomes was first identified in patients with relatively normal renal function [19,20]. Plasma TMAO levels rise as the glomerular filtration rate falls and are approximately 30-fold higher in dialysis patients compared to normal subjects [21]. Several studies of CKD patients with mean estimated GFR between 40 and 50 mL/min/1.73 m^2^ have identified an association between higher TMAO levels and mortality [22,23]. In the HEMO trial, higher plasma TMAO levels in dialysis patients were associated with cardiovascular mortality and time to first cardiovascular event in white patients but not in black patients [24]. Animal studies have demonstrated that increasing TMAO levels cause atherosclerosis in mice [20,25].

Kynurenine, unlike the four solutes described above, is produced entirely by mammalian cell metabolism. It is a branch point of the main pathway of tryptophan catabolism and is converted into products including kynurenic acid, xanthurenic acid, and quinolinic acid [26,27]. It differs from the four microbial derived solutes described above in that its plasma levels do not rise in proportion to the reduction in renal function. Kynurenine has also been associated with serum markers of hypercoagulation in dialysis patients [28]. A recent study identified an association between kynurenine and clotting of the arteriovenous fistula in advanced CKD and in-stent thrombosis in individuals with normal or only moderately reduced renal function [29]. That study further demonstrated that kynurenine upregulated tissue factor in vascular smooth muscle cells and that increasing plasma kynurenine levels in mice promoted clotting in response to vascular injury.

The set of uremic toxins associated with cardiovascular disease described above is not comprehensive and will certainly be expanded as research progresses. It provides, however, a starting point for considering how clearances of potential cardiovascular toxins affect their plasma levels and thus their toxicity.

## 3. Clearances of Potential Uremic Cardiovascular Toxins by the Native Kidney and Their Accumulation in Chronic Kidney Disease

The risk of overall and cardiovascular mortality increases as estimated GFR falls below 60 mL/min/1.73 m^2^ [30]. Plasma levels of all the potential uremic cardiovascular toxins discussed above are elevated at this level of renal insufficiency. Their renal clearances and the degree to which their plasma levels rise with increasing renal insufficiency vary markedly, as summarized in Table 1.

### 3.1. Solutes Cleared by Secretion

All three of the solutes derived from microbial metabolism of amino acids — IS, PCS, and PAG are normally cleared by renal tubular secretion and have clearance rates significantly higher than the GFR. Tubular secretion allows the kidney to clear solutes from the entire renal plasma flow and maintain lower plasma solute levels than would be achieved if clearance were provided by glomerular filtration alone. Among these solutes, the clearance of PAG is easiest to describe. PAG is not significantly bound to plasma proteins, so that clearances expressed in terms of the total plasma solute level and the free, unbound plasma solute levels are practically the same. In individuals with normal renal function, renal PAG clearance is approximately 3-fold higher than creatinine clearance [40]. As CKD progresses from stages 3 to 5, the renal PAG clearance falls along with the GFR and plasma levels of PAG rise [14].

Unlike PAG, IS and PCS are largely bound to albumin so that the free solute levels to which tissues are exposed are a small fraction of the total solute levels [40,48]. In normal subjects, clearances of these solutes expressed in terms of the total solute level are less than the glomerular filtration rate [31]. When expressed in terms of the free, unbound solute level, however, their clearances are not only higher than the glomerular filtration rate but higher than the renal plasma flow. Such high clearance rates can be achieved because the binding of these solutes to albumin is rapidly reversible. As unbound PCS and IS are secreted into the proximal tubule lumen, bound solute dissociates from albumin in blood flowing through the peritubular capillaries, becoming available for secretion. The combination of protein binding and tubular secretion allows free levels of these solutes to be maintained lower than could be achieved by tubular secretion alone. As GFR decreases, renal clearances of IS and PCS decrease and total plasma levels rise in proportion to the estimated GFR [49,50].

TMAO, the microbial metabolite of quaternary amines also likely undergoes secretion in the proximal tubule [42,51]. Reports vary, however, as to whether tubular secretion increases the rate of TMAO clearance above the glomerular filtration rate [21,23,42]. Like plasma levels of PCS, IS and PAG levels of TMAO increase as the GFR falls across CKD stages 3–5 [22,23,41].

### 3.2. Secretion by the Residual Kidney in Advanced Renal Insufficiency

The degree to which the kidney maintains secretory clearance is an important determinant of levels of the secreted solutes IS, PCS, PAG, and TMAO in individuals with advanced CKD and residual renal function. It has often been assumed that glomerular filtration and tubular secretion decline at similar rates. There is growing evidence, however, that for some solutes clearance by glomerular filtration and tubular secretion decrease to a different extent [52]. A recent study reported that the ratio of clearance by tubular secretion to clearance by glomerular filtration changed with estimated GFR for half of drugs that underwent tubular secretion [53]. For most of these drugs, secretory clearance decreased to a greater extent than estimated glomerular filtration. A reduction in the ratio of secretory clearance to glomerular filtration can be explained by the fact that secretory clearance depends on active transport processes that can be inhibited at multiple sites. For instance, decreased expression of secretory transporters has been observed following surgical kidney reduction in rodents [54,55]. Alternatively, retained uremic solutes could saturate transporters and secretory function at lower levels of GFR. Consistent with this theory, multiple uremic solutes inhibit OAT function in vitro at levels observed in CKD [56]. Current evidence is insufficient to establish whether secretion of IS, PCS, PAG, and TMAO is impaired in patients with CKD as GFR falls. A decline in clearance of these solutes relative to creatinine clearance has been reported in CKD [14,49], but this could reflect an altered relationship between creatinine clearance and GFR. Recent studies have identified reduced secretion of PCS relative to estimated GFR in the residual kidney in ESRD [57]. Further work will be needed to clarify the extent to which the secretory clearances of uremic cardiovascular toxins are maintained in advanced CKD.

### 3.3. Mechanisms of Secretion

The molecular mechanisms responsible for tubular secretion of uremic toxins are only partially known. Both PCS and IS are secreted by the organic anion transporters (OAT) 1 and 3 [51,56,58,59,60]. The mechanism of PAG secretion is not known but appears to be independent of OAT1 and OAT3 [51]. TMAO may be secreted in the proximal tubule by both organic cation transporters 1 and 2 and organic anion transporter 3 [42,51]. The organic anion transporters are located on the basolateral membrane of proximal tubular cells and move anions from the plasma into tubular cells up a concentration gradient. This is accomplished by “tertiary active transport” in which the sodium gradient established by Na/K-ATPase promotes entry into the cell of dicarboxylic anions, which are then exchanged for uremic organic anions [59]. Transport of the uremic solutes from within tubular cells into the tubular lumen is less well studied but is thought to be accomplished by a variety of solute carrier families and ATP-binding cassette transporters [59].

### 3.4. Solutes Whose Levels are Not Proportional to Renal Clearance

Kynurenine presents a pattern of accumulation in CKD that is markedly different from the potential uremic cardiovascular toxins, which are normally cleared by secretion. Plasma kynurenine levels are elevated in CKD stage 3 but do not rise further as CKD progresses [36,43]. Of note, kynurenine is not a metabolic end product that is normally excreted by the kidney. It is, rather, an intermediary metabolite, and nearly all kynurenine that undergoes glomerular filtration is reabsorbed by the renal tubules so that very little kynurenine normally appears in the urine [45]. At present, we cannot explain why plasma kynurenine levels rise in renal insufficiency given that kynurenine is not cleared from the body by the kidney. Most kynurenine is produced in the liver, and its production depends largely on the dietary intake of tryptophan. Extrahepatic kynurenine synthesis has been observed in states of inflammation and could contribute to the increase in plasma kynurenine levels in renal insufficiency [26,27]. Alternatively, renal insufficiency could impair conversion of kynurenine to one of its downstream metabolites using an unidentified mechanism.

## 4. Clearance and Accumulation of Potential Uremic Cardiovascular Toxins in Hemodialysis

The accumulation of potential uremic cardiovascular toxins in hemodialysis patients is summarized in Table 1. Total plasma levels of IS, PCS, TMAO, and PAG are typically more than 10-fold higher in pre-dialysis compared to normal control plasma. These levels are generally greater than or equal to levels measured in stage 5 CKD before the initiation of dialysis. The free, unbound levels of IS and PCS are further elevated in pre-dialysis plasma, as the free fraction is higher in hemodialysis patients compared to controls [31].

Characteristics of uremic solutes summarized in Table 1 account for the extent of their accumulation in hemodialysis patients. The levels to which solutes normally cleared by the kidney accumulate in hemodialysis patients depend on two factors [31]. The first is the ratio of their dialytic to native kidney clearance. The second is the extent to which their plasma concentrations are reduced during individual hemodialysis treatments. Solutes for which the dialytic clearance is low relative to the native kidney clearance tend to accumulate to high levels in the plasma of hemodialysis patients. As described above, the native kidney clears IS, PCS, and PAG by tubular secretion, a process which is not replicated by dialysis. Plasma protein binding further reduces the dialytic clearance of IS and PCS. For these solutes, the hemodialytic clearance is low relative to the native kidney clearance. This helps explain why free plasma levels of IS and PCS are much higher relative to normal than urea levels in hemodialysis patients [31]. The ratio of dialytic clearance to native kidney clearance is not as low for PAG and TMAO as it is for free IS and free PCS [21,31]. For these solutes, however, the dialytic clearances are high relative to the volumes of distribution so that the plasma concentrations fall markedly during each hemodialysis treatment. Relatively little solute is therefore removed during the latter part of each treatment, and predialysis plasma PAG and TMAO levels remain elevated far above normal.

The case of kynurenine is again markedly different. Plasma kynurenine levels do not rise progressively as GFR falls and are similar in stage 3 CKD and hemodialysis patients. Kynurenine is removed by hemodialysis, but it is not clear whether removal of kynurenine with dialysis has a notable effect on average plasma kynurenine levels in hemodialysis patients [35,36,61].

## 5. The Effect of Increasing Intensity of Hemodialysis on Plasma Levels of Uremic Cardiovascular Toxins

The effect of increasing the intensity of hemodialysis on plasma levels of potential uremic cardiovascular toxins has been examined in several studies summarized in Table 2. Overall, increasing dialysis intensity has had disappointingly little effect on plasma solutes levels. In the HEMO trial, patients undergoing 3-time-weekly hemodialysis were randomized to standard or high dose dialysis achieved by increasing blood flow rate and treatment time [62]. This change in the dialysis prescription achieved a wide difference in urea kinetics with a single pool Kt/V_urea_ 1.71 in the high intensity group compared to 1.32 in the standard group. There was, however, no difference in overall death or cardiac death between groups. Pre-dialysis levels of IS, PAG, and TMAO were only 7 to 11% lower in the high dose group, while PCS levels were no different [33]. In the Frequent Hemodialysis Network Daily Trial (FHN daily), both the frequency of treatment and total weekly treatment time were increased [63]. There was no difference between groups in death or hospitalization unrelated to vascular access. Pre-dialysis IS and PAG were lower by only 10 and 29%, respectively, in patients receiving treatment 6 times weekly compared to those receiving treatment 3 times weekly, while PCS levels were again no different [34]. In a study of extended duration nocturnal hemodialysis, the duration of thrice weekly treatment was increased from 3.8 to 7 hours [61]. PCS, IS, and TMAO levels were lower by only 5 to 10% in patients receiving long duration treatment, while kynurenine levels were slightly higher.

Several factors may explain why increasing the intensity of hemodialysis has not caused larger reductions in plasma solute levels.

### 5.1. Intermittency of Treatment

Plasma levels of TMAO and PAG are both reduced by approximately 80% during conventional dialysis treatment. Increasing the clearance of solutes with such high reduction ratios cannot reduce their plasma levels much further. Additionally, increasing treatment duration will not reduce solute levels unless treatment is extended over a large part of the day [64]. The intermittent nature of hemodialysis thus limits the ability for changes in dialysis prescription to reduce levels of solutes efficiently removed by conventional treatment. Increasing the frequency of treatment may have a larger effect, but the predicted effect of an increase in frequency from 3 to 6 times weekly while holding total treatment time constant is modest for solutes whose behavior has been modeled.

### 5.2. Changes in Solute Production

Changes in solute production may limit the ability of increased dialysis intensity to reduce levels of some uremic solutes. In particular, the production of PCS may increase in response to an increase in its removal by dialysis. In a crossover study of patients on thrice-weekly nocturnal hemodialysis, a two-week period of increased PCS clearance did not lower plasma levels. Instead, the removal of PCS increased significantly, presumably reflecting an increase in PCS production [65]. In a study of peritoneal dialysis patients, residual renal function accounted for the majority of PCS and IS removal when it was present [32]. Plasma PCS levels were, however, only slightly higher in anuric patients compared to patients with residual renal function. Instead, daily solute removal was much lower in the anuric patients, presumably reflecting lower solute production. These studies suggest that feedback mechanisms may limit the accumulation of PCS and perhaps other uremic solutes, so that increases in solute production offset increased removal when the intensity of dialysis treatment is increased.

### 5.3. Non-Renal Clearance

Non-renal, non-dialytic clearance is another factor that can limit the ability of increased dialysis intensity to reduce plasma solute levels [64]. Non-renal clearance operates continuously. Even a low level of non-renal, non-dialytic clearance can therefore remove a larger portion of solute than a dialytic clearance applied intermittently [66]. Such non-renal clearance has so far been most clearly demonstrated for low molecular weight proteins. For example, β_2_ microglobulin has a non-renal, non-dialytic clearance around 3 mL/min [67]. In the HEMO study, patients in the high flux arm had a 10-fold higher dialytic clearance of β_2_ microglobulin but only a 20% lower plasma level [68]. The presence of a continuous, non-renal, non-dialytic clearance likely explained the failure of higher dialytic clearance to produce a greater reduction in plasma β_2_ microglobulin levels in HEMO. This non-renal clearance likewise presumably accounts for the failure of hemodiafiltration to lower β_2_ microglobulin levels much below those seen with hemodialysis [69]. Clearance studies employing labeled compounds will be required to determine if smaller uremic solutes have significant non-renal, non-dialytic clearances. To date, no such studies have been made for IS, PCS, PAG, or TMAO. We know that the majority of kynurenine is cleared non-renally by metabolic degradation, but the rate of its clearance from the plasma has not been well characterized [26].

## 6. Clearance and Accumulation of Potential Uremic Cardiovascular Toxins in Peritoneal Dialysis

There is limited data on the peritoneal clearance of potential uremic cardiovascular toxins. The peritoneal clearances of the protein-bound solutes PCS and IS are much lower than the clearances of unbound small solutes [32,38,70,71]. In peritoneal dialysis patients with significant residual renal function, more PCS and IS is removed in the urine than in the dialysate [32,38]. Based on these findings, one might expect PCS and IS concentrations to rise to very high levels in peritoneal dialysis patients who become anuric. Studies of such patients have revealed, however, that their plasma PCS and IS levels do not rise significantly higher than those of anuric hemodialysis patients. A potential explanation for this finding is that PCS and IS production rates fall as their plasma levels rise [32]. Levels of TMAO and kynurenine have also been reported to be about the same in peritoneal dialysis and hemodialysis patients, but the peritoneal clearances of these solutes have not been measured [72,73].

Similar to the finding of the HEMO trial in hemodialysis patients, the Adequacy of Peritoneal Dialysis in Mexico (ADEMEX) trial in peritoneal dialysis patients found that increasing the intensity of dialysis as reflected by an increase in Kt/V urea did not reduce overall mortality or mortality from myocardial infarction [74]. No plasma samples were collected, however, and it is thus not possible to determine whether the lack of benefit was associated with failure to reduce the plasma levels of potential uremic cardiovascular toxins.

## 7. What Can Be Done

As described above, increasing the dialysis dosing parameter Kt/V_urea_ and the frequency of in-center hemodialysis has not notably reduced levels of potential cardiovascular uremic solutes [33,34]. A potential exception is urea, which could contribute to cardiovascular disease by promoting carbamylation [75]. In the HEMO study, however, urea levels were lowered without notable effect on cardiovascular endpoints [62]. Other means of increasing clearance of protein-bound solutes have been devised. Hemodiafiltration can increase the clearance of protein-bound small solutes and low weight molecular proteins such as β_2_ microglobulin [69]. The ultrafiltration rate required to significantly increase the clearance of protein-bound small solutes is greater, however, than the ultrafiltration rates that have been employed in randomized trials comparing the effect of hemodiafiltration with conventional hemodialysis [69,76]. Dialysis with fractionated plasma separation and absorption columns modestly increases removal of p-cresol but is associated with circuit clotting [77]. Increasing dialysate flow and dialyzer size over a period of two weeks was shown to increase the dialytic clearances of IS and PCS but did not significantly reduce their plasma levels [65]. Other means of increasing the clearances of protein-bound solutes include the addition of a sorbent to dialysate and the addition to blood entering the dialyzer of substances, which displace bound solutes or reduce the binding capacity of albumin [78,79,80,81]. All of these methods have been shown to increase bound solute removal during in-vitro dialysis. Clinical studies testing whether they reduce protein-bound solute levels in patients remain to be performed.

It should be noted that increasing solute clearances enough to limit the cardiovascular toxicity of uremic solutes may prove difficult. For instance, an early study of TMAO found that individuals with cardiovascular events had a mean TMAO level of 5 μM, while individuals without events had a mean level of 3.5 μM [19]. In comparison, hemodialysis patients have pre-dialysis TMAO levels as high as 77 μM and post-dialysis levels around 11 μM [21]. Only many hours of treatment with high clearance could reduce TMAO from the high end stage renal disease levels to the much lower levels previously associated with improved cardiovascular outcomes. As described above, plasma levels of PAG, PCS, and IS are also elevated far above normal in patients with renal failure. Until harmful levels of these uremic solutes are better defined, we cannot predict the cardiovascular benefit of increasing their removal by dialysis.

Limited ability to remove potential cardiovascular uremic solutes with dialysis has stimulated interest in inhibiting solute production. The majority of potential uremic cardiovascular toxins we have discussed are produced by colon microbes. Their production by non-mammalian metabolism in an isolated body compartment may be particularly susceptible to inhibition [14,82,83,84,85,86]. Methods that have been employed so far in attempts to reduce the production of microbial-derived solutes include administration of probiotic and prebiotic compounds [69,87,88]. Ultimately, it may be possible to install in renal failure patients a genetically engineered microbiome that does not produce toxic solutes.

Further development of methods to reduce levels of uremic cardiovascular toxins will require better understanding of the mechanisms of solute production and clearance. It should be emphasized that mechanisms of reducing solute levels will be different for different solutes. The solutes we have focused on in this review may serve as an example for other potential uremic cardiovascular toxins. Indoleacetic acid, for instance, shares many characteristics with IS and PCS. Indoleacetic acid is derived from the action of colon microbes on tryptophan and is protein-bound in the plasma. Indoleacetic acid levels my thus potential be reduced by manipulation of the colon microbiome or by measures which increase the clearance of bound solutes. Asymmetric dimethylarginine (ADMA) shares many characteristics with kynurenine. ADMA is formed in mammalian cells and is cleared largely by enzymatic degradation [89]. Levels are elevated in CKD stage 3 and are not notably higher in ESRD patients on hemodialysis [90,91]. Reduction of ADMA levels may require manipulation of metabolism in mammalian cells. Given the variety of potential uremic cardiovascular toxins, development of treatments to comprehensively lower their levels is a daunting prospect.

## 8. Conclusions

Uremic solutes likely contribute to cardiovascular disease in renal insufficiency. Most but not all of the solutes that have been associated with cardiovascular disease are eliminated by the native kidney through tubular secretion. Levels of these solutes rise as CKD progresses and are even higher in the plasma of dialysis patients than in individuals with advanced chronic kidney disease. Increasing the intensity of dialysis has not notably reduced plasma levels of these solutes or improved cardiovascular outcomes. The ability to reduce plasma levels of uremic cardiovascular toxins by increasing dialysis intensity may be limited by the intermittency of hemodialysis, by changes in solute production, and by the presence of non-renal, non-dialytic clearance. The limited effect of increasing dialysis intensity has motivated efforts to inhibit the production of toxic solutes.

## Figures and Tables

**Table 1:** Plasma Levels and Clearances of Potential Uremic Cardiovascular Toxins.

	Total Plasma Levels (mg/dL)					Ratio Hemodialysis/Normal		Reduction Ratio with Hemodialysis (%)	Hemodialytic Clearance/Normal Kidney Clearance	Normal Kidney Fractional Clearance	Free Fraction (%)
	Normal	CKD III	CKD V	Peritoneal Dialysis	Hemodialysis	Total Plasma Levels	Free Plasma Levels				
Urea Nitrogen	7–15	–	–	51–61	45–63	3–5	–	66–75	4.2	0.5	–
	[21,31]			[32]	[21,31,33,34]	[21,31]		[21,31,35,36]	[31]	[31]	
Indoxyl Sulfate	0.1	0.2	0.8	1.6–3.4	1.4–2.9	14–30	116	36	0.21	14	2–16
	[31,37]	[37]	[37]	[32,38]	[31,33,39]	[31,37]	[31]	[31]	[31]	[40]	[37,40]
p-Cresol Sulfate	0.3	1.0	3.3	0.9–2.7	2.1–3.7	7–13	41	31	0.39	7	3–6
	[31,37]	[37]	[37]	[32,38]	[31,33,37,39]	[31,37]	[31]	[31]	[31]	[40]	[31,37]
PAG	0.05	0.2	0.5	–	4.1–5.2	59	122	80	0.37	3	78
	[31]	[14]	[14]		[31,33,34]	[31]	[31]	[31]	[31]	[40]	[40]
TMAO	0.02–0.04	0.06–0.1	0.6	–	0.5–0.8	12–39	–	86	0.75	1–1.8	100
	[21,41]	[23,41]	[41]		[21,23,33,41]	[21,41]		[21]	[21]	[21,23,42]	[21]
Kynurenine	0.03–0.05	0.07–0.1	0.1	0.04–0.1	0.06–0.1	1.8–2.7	–	22–30	–	<0.01–0.1	10–45
	[28,36,43,44]	[36]	[36]	[28,44]	[28,36,43,44]	[28,36,43,44]		[35,36]		[45,46]	[46,47]

PAG: phenylacetylglutamine; TMAO: trimethylamine N-oxide; hemodialysis plasma levels represent pre-dialysis levels; reduction ratios are calculated from pre- and post-dialysis total plasma levels. Urea reduction ratios were 66–75% in referenced studies; hemodialytic clearance/normal kidney clearance is the ratio of mean clearances calculated in terms of free, unbound solute levels for IS, PCS, and PAG and in terms of total solute levels for TMAO; Fractional clearance is ratio of solute clearance to creatinine clearance calculated in terms of free, unbound solute levels for IS, PCS, and PAG and in terms of total solute levels for kynurenine and TMAO.

**Table 2:** Levels of Potential Uremic Cardiovascular Toxins in Trials of Increased Intensity of Hemodialysis.

	HEMO [33,62]			FHN Daily Trial [34,63]			Extended Duration Nocturnal Hemodialysis [61]		
	Standard		Increased Intensity	Standard		Increased Intensity	Standard		Increased Intensity
N	643		638	53		30	20		33
HD treatments per week	3		3	3		6	3		3
Hours per treatment	3.2		3.7	3.6		2.4	3.8		7.0
Length of intervention	34 months			12 months			12 months		
Measure of dialysis intensity	Single pool Kt/V urea			Weekly standardized Kt/V urea			Urea reduction ratio (%)		
	1.32 ± 0.09		1.71 ± 0.11	2.49 ± 0.2		3.54 ± 0.56	77 ± 6		84 ± 8
Clinical outcome	No difference in death, cardiac death, or composite of cardiac death and first cardiac hospitalization			No difference in composite of death or hospitalization unrelated to vascular access			None studied		
	Plasma Total (mg/dL)		Relative Difference (%)	Plasma Total (mg/dL)		Relative Difference (%)	Ratio Final to Baseline		Relative Difference (%)
	Standard	Increased intensity		Standard	Increased Intensity		Standard	Increased Intensity	
Urea nitrogen	63 ± 19	57 ± 18	−10	54 ± 14	45 ± 16	−28	1.06	0.92	-13
Indoxyl Sulfate	2.7 ± 1.3	2.4 ± 1.1	−11	2.9 ± 1.1	2.5 ± 1.0	−10	1.16 ± 0.73	1.1 ± 0.43	-5
P-Cresol Sulfate	3.3 ± 1.7	3.4 ± 1.7	2	3.2 ± 1.4	3.3± 1.6	16	1.14 ± 0.66	1.02± 0.12	-11
Phenylacetylglutamine	4.6 ± 3.0	4.3 ± 2.6	−7	4.4 ± 2.3	3.3 ± 1.6	−29	-	-	-
TMAO	0.80 ± 0.49	0.73 ± 0.49	−9	-	-	-	1.13 ± 0.49	1.07 ± 0.55	-5
Kynurenine	-	-	-	-	-	-	0.93 ± 0.31	1.07 ± .34	15

Values are mean ± standard deviation; TMAO: Trimethylamine N-oxide; HD: hemodialysis. HEMO plasma total values are from predialysis samples collected 3–8 months into intervention. Relative difference calculated from ratio of plasma total levels of increased intensity vs. standard groups during intervention. FHN plasma total values are from predialysis samples collected 12 months into intervention. Relative difference calculated from mean end/baseline ratio in increased vs. standard groups. Extended duration nocturnal hemodialysis samples collected predialysis 12 months into intervention. Relative difference calculated from mean end/baseline ratio in increased vs. standard groups.
